# Neurology

**Published:** 1904-10-01

**Authors:** 


### Progress in Medicine and Surgery.

### NEUROLOGY.

*(Concluded from Vol. XXXVI., page 449.)*

**Migraine.**—In an article on this subject Styll[7] says that Migraine is distinguished from other forms of headache by its sensory accompaniments, its paroxysmal character, definite course, and unilateral distribution of pain. From epilepsy with visual aura it is distinguished by the length of the aura. The treatment of the paroxysm is to put the patient in a darkened room, wash out the stomach with hot water or direct the patient to drink large quantities of hot water. This should be followed by a saline cathartic. Some of the following drugs should be used—antipyrin, phenacetin, bromides, hyoscyamine, ergot, caffein, or nitroglycerin. Morphia should not be given unless the patient is prostrated by the pain. In the intervals, the gastro-intestinal tract should be put into good order and the diet regulated. Ocular defects should be corrected. Out of door life and exercise are invaluable aids. Of all drugs nitroglycerin and cannabis indica are the best. Where there is conspicuous pallor and the pulse tension is increased nitroglycerin should be given; it will often stop the attacks. In the great majority of cases cannabis indica is the most satisfactory remedy, being not merely palliative but curative. It may be given as follows: zinci phosphid., gr. i.; ferri redacti, gr. i.; ext. nux. vom., gr. i.; ext. cannabis indic., gr. i.; m. ft. pil. i.; ter die. The author gives such a pill for a month, then alternates it with some other nerve tonic for a fortnight, after which he resumes the pill for another month, and so on.

**Thrombosis of the Cavernous Sinus.**—Cases of this rare disease have been recently recorded by D. Wight and Germain[8] and by Roth.[9] The infective agent is usually a staphylococcus or a streptococcus, and the primary lesion is usually in the face—furuncle, carbuncle, cellulitis, erysipelas, dental caries or alveolar abscess. Phlebitis develops in the infected region and spreads up to the cavernous sinus, causing thrombosis, which rapidly spreads to the sinus on the opposite side or to the other basal sinuses. The thrombus breaks down and infected particles are carried into the general circulation, producing pulmonary infarction and pyæmic abscesses. Meningitis and cerebral abscess may develop by contiguity. In addition to the general signs of pyæmia, there are local ones—headache, vomiting, convulsions, delirium and a terminal coma. Stasis in the ophthalmic veins produces swelling of the eyelids and ecchymosis and œdema of the skin and conjunctiva on the affected side. Proptosis follows, due to the same cause. Photophobia, pain in the eye, and amblyopia may occur. Some meningitis is almost constant, but a differential diagnosis between thrombosis and meningitis may be made by the ocular and pyæmic symptoms. The prognosis is very bad, most cases being fatal. Treatment is unsatisfactory, for the focus in the cavernous sinus cannot be reached.

**Tumour of the Pituitary Body.**—Cestau and Halberstadt[10] have published a case of a tumour of the pituitary body in a man aged 60 years, in which the symptoms consisted of epileptic vertigo, progressive enfeeblement of memory and intelligence, headache, and marked somnolence interrupted by occasional attacks of agitation. He eventually died after a series of attacks of dyspnœa. There were no acromegalic or osteo-hypertrophic changes, but there was marked adiposity. The necropsy showed a tumour—a cystic adenoma—practically limited to the sella turcica. The important points in this case were the absence of skeletal changes, and the presence of mental changes, such as apathy, enfeeblement of memory and intelligence, and somnolence. The authors suggest that when pituitary tumours occur in infancy they produce gigantism; in early adult life acromegaly; while in old age they cause no skeletal changes but mental symptoms of apathy and somnolence combined with obesity.

**Reversals of Habitual Motions.**—Weir Mitchell has recorded cases in which the chief characteristic was a tendency to reverse normal actions. The patient either did exactly the opposite to what he intended to do, or else did what he wished to do in the reverse way to what it would usually be done. Thus in the street, if the action of walking ceased to be automatic and he recalled the fact he was walking, he would sometimes walk backwards a few steps, and, finally recovering himself, would go on as before. Another patient used to say exactly the reverse of what he meant, so that when he wished to remain in-doors he would say, "I will remain out-of-doors." Pick thinks that such cases may be divided into two classes, those in which there are disturbances of conception and the conception leads to contrary action, and those in which the disturbances seem to be purely motor and in which the reverse motion is substituted for the desired one.

**Senile Paraplegia** occurs in patients over 50 years of age, and is characterised by weakness of the legs with slowness of movements. There is no wasting and no affection of sensation. The knee jerks are normal and ankle clonus is absent. The onset is gradual and the symptoms slowly progressive, but the loss of power is seldom so great as to prevent the patient standing. Similar cases are met with in which the reflexes are increased. The pathology of this affection has recently been investigated by Pic and Bonnamour,[12] who find a general atheroma, and around the arteries of the cord a diffuse sclerosis, not systematic, but with a marked predominance in the crossed pyramidal tracts and in the posterior columns. It is probable, but not clearly proved, that this diffuse interstitial myelitis is of vascular origin.

[8] Boston Med. and Surg. Jour., May, 1902. [9] New York Med. Jour., Feb. 27, 1904. [10] Lancet, Feb. 13. [11] Jour. of Nerv. and Ment. Dis., April, 1903. [11] Ibid., January, 1904. [12] Revue de Med, 1904.

